# Is Tolerance Liberal? Javed Ahmad Ghamidi and the Non-Muslim Minority

**DOI:** 10.1177/0090591720956590

**Published:** 2020-09-11

**Authors:** Humeira Iqtidar

**Affiliations:** 1 King’s College London, London, UK

**Keywords:** modern Islamic thought, tolerance, Javed Ahmad Ghamidi, decolonizing political theory, nonliberal tolerance

## Abstract

Tolerance is claimed not just as central to liberalism, but increasingly as the sole preserve of a liberal order. This essay opens up a critical space for examining the naturalized relationship between liberalism and tolerance by focusing on the political thought of Javed Ahmad Ghamidi (1951–), a prominent Pakistani public intellectual who is often labeled as a “liberal” Islamic thinker. Ghamidi has never identified himself as one. Using as an investigative opportunity the disjuncture between his self-identification and how his ideas are labeled, and placing Ghamidi’s ideas within the wider tradition of Islamic thought, this essay elaborates on his vision of non-liberal tolerance predicated on individual responsibility infused with humility and shari’a-inspired state minimalism. Insight into the depth of nonliberal conceptions can facilitate a reconsideration of the relationship between liberalism and tolerance.

## Introduction

Writing in 1993, Stephen Kautz made the strong claim that “[t]olerance is a liberal virtue: it is among the most honourable of the respectable habits of liberal citizens.”^1^ A decade later, Chandran Kukathas took this association further by arguing that the “value which is fundamental to liberalism is toleration. A society or community is a liberal one if, or to the extent that, it is tolerant.”^2^ Kukathas is an immensely persuasive advocate for a vision of tolerance that is in large part peaceful coexistence through indifference to difference. I use the term tolerance here to signal arrangements for peaceful coexistence in a plural society. There is, of course, some variation in how tolerance is conceptualized by liberal theorists.^3^ I take these debates to be indicative of concerns regarding its precise role in a liberal polity and generative of further questions. We can, however, readily see that an increasingly close association of tolerance with liberalism has become the common sense of post–World War II Anglo-American political theory and, importantly, popular political discourse. Many have seen the liberal ideal of freedom as tied in fundamental ways to tolerance.^4^ Indeed, liberals “are frequently defined as people who value liberty, and the toleration necessary for the promotion of liberty.”^5^

This association is even stronger in mass political imagination. In popular discourse, the two words are used almost interchangeably such that to be liberal is to be tolerant, and to be tolerant is to be liberal. In other words, there is an appropriation of diverse forms of life as liberal if tolerant in practical implications. This is true of a sundry range of ideas and practices from inclusive Sufi praxes in contemporary South Asia^6^ to sexual mores in Africa.^7^ However, the colonization of tolerance by liberalism, its arrogation through the claim that what is tolerant is ultimately liberal, needs interrogation because accepting without challenging the claims of liberal theory blinds us to the depth of nonliberal conceptions of tolerance that may provide viable alternatives. My aim here is modest. Through a detailed look at a nonliberal conception of tolerance articulated by the influential contemporary Islamic thinker Javed Ahmad Ghamidi, I pry apart the close relationship between liberalism and tolerance, allowing critical consideration of the specificity and limitations of liberal tolerance, and display the contours of a nonliberal, but not antiliberal, conception of tolerance.

In the contemporary political context, there is a heightened enthusiasm for locating liberal impulses within Islam, given the increasing association of intolerance and extremism with it. Hailed in an American newspaper as “a bit of a rock star—adored, hated, popular, and notorious all at once,”^8^ Ghamidi (1951–) has been hard to label. He has been called a “critical traditionalist”^9^ as well as a liberal reformer.^10^ Central, for those who claim him as “liberal” is his opposition to Islamist visions of political responsibility, as well as the idea that he articulates a conception of a tolerant society where the state does not impose norms of correct behavior and where difference, particularly religious difference, does not preclude peaceful coexistence. Interestingly, Ghamidi himself has never identified with the label of a liberal thinker,^11^ nor does he use the term tolerance or attempt a translation of it. In fact, he has explicitly argued against using a liberal lens to understand Islam, claiming that such a move will inevitably limit our understanding of shari’a.^12^ Liberalism, for him, is an ideological framework promoting individual rights that is tied closely to Europe’s historical development and that, he proposes, needs assessment from an Islamic perspective. Most importantly, his ideas are not a “pale reflection” of European ideas, a charge leveled against many modern Islamic thinkers.^13^ Rather he draws inspiration explicitly from Islamic history and Quranic injunctions to revive what he sees as an Islamic norm of tolerance. Taking precisely this difficulty in categorizing his ideas as a generative starting point, I argue that Ghamidi’s vision of state, society, and individual is indeed tolerant, but it is not liberal. Drawing upon his reading of Quranic ideals and Islamic historical experience, Ghamidi articulates a vision that is neither reliant upon the notion of individual freedom of conscience nor does it rest on the advocacy of individual rights. Instead, in his work, there is an emphasis on individual responsibility that is infused with humility and shari’a-inspired state minimalism as the preconditions for fostering tolerance. Tolerance is a duty, not a right, in his vision.

That there are different histories linked to Islamic conceptions of peaceful coexistence from the historical experiences that gave rise to liberal tolerance is an important reminder. Liberal tolerance is a product of European history where religious difference emerged as a fundamental threat to the sovereignty of the state.^14^ Many other traditions of thought and practice, including the Islamic, did not place such emphasis on religious homogeneity within the polity. Recognizing historical and philosophical differences is productive in opening the space for serious consideration of alternative futures that might address the limitations of liberal visions. The purpose here is quite explicitly *not* to reconcile the two traditions of thought and practice. This, however, does not mean that the interest is in articulating an antiliberal stance; there is an important difference between nonliberal and antiliberal. It is, of course, entirely possible to “pose similar questions across philosophical and ethical traditions with the aim of identifying the possible grounds for consensus” and to find overlaps, as March^15^ has done in a nuanced study of Islam and liberal citizenship. However, such inquiries into compatibility tend to constitute an epistemic colonization of tolerance because they shield liberalism’s premises from challenge, and in so doing confirm liberalism’s primacy as the normative standard in terms of which Islamic thought (and any other expression of political thought) is appropriately judged.^16^ This is one of the central and insidious forms by which tolerance is sutured to liberalism.

In contrast, evaluating ideas and practices in their differences is an important strategy for thinking through what David Scott has called “alternative postcolonial futures” that “reject the view that it is possible—or even desirable—to arrive at a single political principle that all forms of life ought to subscribe to, and . . . to affirm the validity of a variety of moral traditions and forms of political reasoning which, however reconfigured by modern power, produce the varied goods of human flourishing.”^17^ The overarching interest here, then, is in opening a discussion about such alternative futures that move past liberalism’s Eurocentric reading of individual right to freedom of conscience as foundational to tolerance and engaging with other modes of peaceful coexistence that carry widespread historical and normative resonance.

In the first section, I provide an introduction to Ghamidi’s ideas regarding what he calls “political” shari’a to lay the ground for an exploration of his ideas regarding minorities in his ideal state. In the second section, I explore two key elements of his ideas related to tolerance: state minimalism and pious responsibility infused with humility. The final section opens up the seemingly close relationship between tolerance and liberalism based on these discussions of Ghamidi’s vision and alerts us to the possibility that the colonization of tolerance by liberalism may occlude other viable and capacious notions of tolerance that are in closer accord with the lived experience of different religious and historical traditions.

## Javed Ahmad Ghamidi: Tolerant Thus Liberal?

Javed Ahmad Ghamidi, a Pakistani religious scholar, Quranic exegete, and public intellectual, established a presence for himself through the publication of his key contributions as an exegete—*Al-Burhan* (1993) and *Al-Mizan* (1990)—and continues to play an important role in Pakistan’s intellectual landscape through his publishing, TV shows, research and teaching institute (*al-Mawrid*), and school system (*Mus’ab* schools). While he studied philosophy and English literature at university level, his decision to write in Urdu seems motivated by his desire to reach a wide audience across Pakistan, India, and Bangladesh. English translations of his work are routinely published by the *al-Mawrid* Institute he founded, and Ghamidi has a sizeable following in South and South-East Asia, as well as Australia, the United Kingdom, and the United States. The establishment of The Ghamidi Center of Islamic Learning in Dallas, Texas, in 2019 signals this international presence. Despite, or perhaps, because of this influence, a noticeable body of *ulema* (religious scholars) continues to oppose him on questions of methodology and interpretation.^18^

Ghamidi has proposed a very structured and demanding methodology for interpreting shari’a, the normative framework of Islam. Trained in the Hanafi school initially, Ghamidi advocates a direct engagement with the Quran, supplemented primarily by literary and linguistic contextualization from pre-Islamic and early Islamic Arabia, rather than building upon *fiqh* literature that discusses the philosophy and general principles for interpreting the Quran and the tradition of the prophet. Deepening the hermeneutic rules for Quranic interpretation developed by his mentors, Maualana Amin Ahsan Islahi (1904–1971) and Maulana Hameed-ud-Din Farahi (1862–1930), and reflecting a trend that goes much beyond the Salafi rejection of *fiqh* literature, Ghamidi has systematized the Quran on the basis of some variables including the context of revelation of a verse, its thematic focus, and its addressee. He has built upon the notion of *nazm* or coherence of the Quran that Farahi first proposed in detail and that Islahi developed further. Bringing a similarly systematic approach to the study of *sunnah*, which he defines as the lived experience of *din* (Islamic way of life), Ghamidi lays out specific principles to sharply structure the manner in which these might be understood.^19^

Building on his methodological principles, Ghamidi advocates a highly restricted role for individual interpretation of the Quran. His approach might look simple due to its disregard for the vast and complicated *fiqh* literature, but, in fact, relies heavily on deep immersion in history, philology, literature. and philosophy of early Islamic life. Following his own general guidelines, Ghamidi establishes a strictly minimalist role for the state while at the same time exhorting Muslims to “fully cling to state authority in all circumstances.”^20^ In his elaboration of shari’a he claims that individuals do not have the right to challenge the authority of the state. Indeed, even in the worst circumstances,

no Muslim citizen has been given the permission to revolt against the government unless he has the backing of a clear majority behind him . . . if the majority does not support him, then such a revolt would not be against the government; on the contrary, it would be against other Muslim citizens, which according to the shariah is *fasad fil ard* (spreading lawlessness and anarchy in society)—an offence regarded by the Quran as punishable by death.^21^

This is a direct intervention against the more aggressive Islamist interpretations of the political responsibility of individual Muslims. Here Ghamidi is building on a major concern within Islamic thought—stability of the political realm. Revolt without majority support is bound to lead to anarchy or civil war, an anathema to Islamic political thinking.^22^ In contrast, a majority support gives assurance that the revolt will succeed while achieving a warranted political change, precisely because a majority is behind it. Ghamidi’s concern for stability carries within it an implicit acknowledgement that a successful change of rulership, violent or not, invariably commands for itself the right to political sovereignty. A violent change can allow claims to sovereignty even if divorced from the interests of the majority or any moral framework. For Ghamidi, such a polity is not likely to be oriented toward justice, the lodestar of Islamic theories of rulership.^23^

However, rulers bear a heavy responsibility—that of ensuring that justice prevails in the polity. The responsibility to govern with justice is a demanding one that should only be undertaken by those who realize the obligation it places upon them. Building on his teacher Amin Islahi’s work, Ghamidi argues that the burden is all the more onerous for pious rulers because they would recognize that “even the most concealed injustice is in His knowledge.”^24^ Given how much is asked of rulers in an Islamic polity, according to Ghamidi’s interpretation, those who truly recognize the weight of their leadership role would naturally be reluctant to take it on. Relying on the prophet Mohammed’s sayings recorded in the authoritative eighth-century collections by Imam Bukhari and Imam Muslim, Ghamidi suggests that the best candidates for the positions of rulership are those who do not seek the post and are compelled due to their piety to fear the accountability it entails. This idea has a long and important lineage in Islamic theories of rulership.^25^

The main responsibility that citizens have in Ghamidi’s ideal polity is to choose the right people to govern over them. This is not an easy task, of course. Ghamidi interprets the Quranic verse 42:38 “and their system is based on their consultation” to mean that,

the system itself be based on consultation; everyone should have an equal right in consultation; whatever is done through consultation should only be undone through consultation; everyone that is part of the system should have a say in its affairs and in the absence of a consensus, the majority opinion should decide the matter.^26^

Ghamidi builds on the argument laid down by his erstwhile teacher, the Islamist Abul A’la Maududi,^27^ to explain how consultation has to be built in at a systemic level. Maududi argued, and despite other differences Ghamidi agrees, that the people whose interest and rights relate to any issue should first have freedom to know the details of matters requiring decisions, to criticize and to change their rulers if the faults are not rectified. Second, consent to rule has to be obtained without force, intimidation, greed, or deception. Third, the people chosen for consultation should enjoy the confidence of the majority. Those consulted (i.e., those who are part of the consultative body or *shura*) should be free to express opinions based on their knowledge and conscience, otherwise it would be a negation of the principle of consultation. Finally, and critically, a decision made through

the consensus or majority opinion of the members of the *shurā* or which has the mandate of the people behind it must always be accepted. The Almighty has not said: “they are consulted in their affairs”; on the contrary, He has said “their system is based on their consultation”. Merely consulting people does not fulfil this directive; it is necessary that a consensus or majority opinion be considered as decisive in running the affairs.^28^

Once the citizens of an Islamic polity have chosen their leaders in this manner, the state can ask very little of its citizens. Muslim citizens can only be asked to fulfil their responsibilities as Muslims in two main ways—that they agree to pray and to pay *Zakat*, an annual tax on wealth. All are equally entitled to the rights of citizenship without prejudice. There is no injunction, according to Ghamidi, for Muslims to try and judge whether one group or individual is a proper Muslim or not. The state has no right to enforce any practices.^29^

In making these arguments Ghamidi rejects, without naming directly, some versions of Islamism and, more specifically in Pakistan, the laws and directives imposed during General Zia’s reign (1978–1989), a military dictator supported through US military aid and political sponsorship during the first Afghan war (1979–1990). General Zia’s Islamization program, inspired in part by Maududi’s support for state-led social transformation, entailed state-enforced closure of restaurants during *ramzan*, the month of Muslim fasting; an emphasis on collective prayers in all public organizations; and a regime of public morality through legal and extralegal mechanisms to support conservative clothing, gender discrimination, prohibition of alcohol and gambling, and aggressive policing of cultural production. Moreover, Zia’s regime strengthened discriminatory anti-Ahmadiyya^30^ legislation that barred this particular group from calling themselves Muslims. As mentioned above, Ghamidi argues, using historical and theological arguments, that according to shari’a none of this is within the remit of the state to enforce. These are all issues related to the individual’s relationship with God, to be resolved by each individual privately.

In similar vein, he criticizes the government of Iran and Saudi Arabia to argue that state laws cannot be made to distinguish between Muslims on the basis of their piety or to impose aspects of shari’a that are clearly a pact between the individual and Allah, and not the state and the individual. Thus, he said, when in Saudi Arabia women were prohibited from driving, this was clearly not in line with the Quran,^31^ not because cars were not invented when the Quran was written as some commentators had suggested, but because the state cannot make such demands on its citizens.^32^ As before he argues that Allah’s claims on Muslims are separate from the claims of the state. The collective arrangement (*nizam-e-ijtemai*) can only demand from Muslims three things specified earlier: choosing the rulers actively, prayers, and zakat—nothing beyond that.^33^

The discursive emphasis on Islam within Pakistan under General Zia’s regime also exerted immense pressure on Pakistan’s non-Muslim citizens to establish their credentials as citizens. Ghamidi supports the state’s right and duty only to distinguish between Muslim and non-Muslim citizens but in a way that ultimately undermines the value of such a distinction. Ghamidi proposes that the constitution of Medina after the prophet Mohammed established a state there meant that Jews and Muslims were equal citizens once they accepted the political leadership of the prophet Mohammed.^34^ He goes on to suggest that the verse in the Quran that seems to imply that non-Muslims must pay *jizyah*, while the Muslims pay *zakat*, refers only to such a group of non-Muslims to whom a perfect messenger in the form of Mohammed had proselytized. Since later non-Muslims have not had the benefit of learning from such a perfect embodiment of Islamic behavior, the verse does not apply to them.^35^

By making this argument, Ghamidi effectively takes away the option of treating non-Muslim citizens any differently from the Muslim ones by eliminating the one key marker of their distinction—a different system of taxation. As a general rule, *zakat* is 2.5% of long-term assets but 10–12.5% on agricultural produce. There is no clear minimum or maximum amount for *jizya*, although the scale was often graded such that the poor, sick, minors, monks, and dependents did not pay.^36^ Historically there seems to have been significant variation including *jizya* levied at a lower, as well as higher, rate than *zakat*.^37^ Ghamidi argues that any agreement is possible with non-Muslim citizens of an Islamic polity regarding their rights, keeping in mind “the circumstances and the various international accords one is bound with.”^38^ This is consistent with the various agreements struck with different conquered populations in early Islamic history, many of which were the continuation of pre-Islamic Byzantine and Sassanid practices.^39^ In positing the possibility of a range of options in terms of agreements that the state would make with its non-Muslim citizens, Ghamidi is arguing against those who make a case for a clear hierarchy of citizenship in an Islamic polity, as well as those Islamists who do not recognize this implication of their arguments for such distinctions.

In a perceptive study, Anver Emon has argued for moving beyond the details of the rules to thinking through why the *dhimmi* rules, regarding norms of inclusion for non-Muslims,^40^ existed at all. He suggests that premodern jurists “did not simply recognize but rather seemed to take for granted the fact of diversity in a Muslim polity. The issue for them was not whether non-Muslims should or should not reside under the Muslim imperium. Rather, the issue was how best to regulate their presence in a public sphere defined in Islamic terms . . .” such that the core values of Islamic governance were not adversely impacted.^41^ Ghamidi too focuses on the philosophical role of *jizya*, and *dhimmi* rules to frame his argument and attempts to take contemporary debates back to that past Islamic sensibility when inclusion of difference was treated as the norm rather than the exception, in contrast to the long history of eradication of religious difference in the European context. Ghamidi’s vision appeals to many as a theologically sanctioned seemingly secularized Muslim state—one that is led by Muslims but does not attempt to enforce norms of behavior upon its citizens.^42^ While the state takes on the burden of some weighty decisions such as when to wage war, it is otherwise quite removed from everyday life and certainly has no right to interfere in the regulation of personal and communal life.

## State Minimalism and Individual Humility

At first glance, Ghamidi’s tolerant approach seems to merge smoothly with the tolerance liberalism has claimed for itself—a framework that establishes strict limits to the use of state coercion, promotes a laissez-faire attitude toward religious minorities, and encourages the establishment of clear rules of engagement between the state and a quietist citizenry. Contemporary discourses of liberal tolerance link all of these to the fundamental principle of individual freedom and rights.^43^ However, Ghamidi’s tolerance is independent of the question of rights, and individual freedom does not provide the rationale for his minimalist state. His state is minimalist in keeping with shari’a philosophical and historical norms where vast domains of social and individual life are not imagined to be within the purview of the state. His citizens are tolerant due to their pious humility and responsibility, making them reluctant to pass judgment on others. Theirs is not a position of moral relativism but of following a moral code that requires them to defer final judgment of individual actions to Allah.

In Islamic thought, in general, difference is taken as a given, building on the Quranic statement that “if Allah had wished, Allah could have made all humans into one community” (5:48), as Ghamidi often reminds his audiences.^44^ That human beings will divide themselves into nations, tribes, or other kinds of groups is an expression of Allah’s will and is a long-running element of Islamic philosophy and historical practice.^45^ Islamic thought has taken not just substantive difference of argument and perspective to be a given but has also institutionalized difference as an integral aspect of the Islamic scholarly and legal tradition through the different schools of shari’a interpretation.^46^ The exact contours of what was seen as an acceptable range of differences varied over time and space, but the principle of difference in interpretation as valid was clearly established. Islamic historical experience until to the nineteenth century indicates a lack of will and legitimacy in eliminating religious difference beyond specific and often notable exceptions,^47^ unlike the European experience of institutionalized questioning of individual conscience through the Inquisitions and long-running wars of religion that had a foundational impact on the formation of the modern European nation-state.^48^ Nevertheless, this lack of interest in homogenization is not the same as a celebration of diversity. It is, of course, not clear that despite extensive accommodation of other faith communities Muslims necessarily took particular pride in it or saw it as a specific requirement of their religiosity rather than just an accepted part of the world they inhabited.^49^

For Ghamidi, religious diversity coupled with state minimalism has been an integral part of shari’a as lived experience. In holding this view he is supported by important recent scholarship; the long history of shari’a is remarkable precisely as a self-enforcing mechanism where imposition by the state was not central to its functioning.^50^ The *ulema* have, for the most part of premodern and modern Islamic history, not represented the state, nor have their scholarly rulings or *fatawa* (sing. *fatwa*) been backed by state imposition. Unlike the European experience prior to and through the Reformation,^51^ they have prized their distance and independence from the state.^52^ Contemporary Euro-American scholarship on tolerance in Islamic thought and practice has built on this historical experience and philosophical predisposition to suggest that Islam is compatible with liberal tolerance^53^ or that the resources for significant overlap are available.^54^ These are helpful perspectives; however, it is also useful to recognize the differences and to think about their implications.

Ghamidi is trying to recapture that spirit of moral self-enforcement that is not reliant upon state imposition and a sense of selfhood that prizes individual duty rather than rights, as well as humility in sociopolitical life. Thus, his reasons for advocating a somewhat passive state that stays away from vast domains of social life are very different from those for whom the shape of the state is inevitably tied to the issue of individual freedom of conscience that is central to liberal tolerance.

Islamic thought takes a form of freedom of conscience as a given, enshrined in the notion of *niyyat* or intention defining all acts.^55^ The centrality of *niyyat* in social life as well as legal discourses balances the notion of divine predestination with free human will to choose between multiple courses of action. Islamic legal debates on *niyyat* assume and engender spiritual equality. Ghamidi’s arguments stem not from secular humanism that is thought to be fundamentally entwined with liberal tolerance ^56^ but from a continued acceptance of the superiority of the divine and individual humility in the face of it.

This requires some explanation since humility is seen as a problem in an important strand of post-Enlightenment European thought because it is believed to be linked closely to servility. As Button has perceptively noted in his defense of humility, cultivation and acceptance of lowliness is, for many, a fundamental stumbling block within a liberal polity, for “if humility entails the acceptance of a low position for oneself as what is one’s due, then humility would seem to be a vice for liberal citizens committed to the equal moral standing of persons and for individuals.”^57^ This discomfort with humility in Euro-American political theory is linked to its uses in European Christian theology following Thomas Aquinas and the association with meekness. Ghamidi is, however, deploying a different conception of humility, one that is infused with a deep sense of responsibility for one’s actions that does not rest on comparison and condemnation of the actions of others.

Ghamidi argues that human beings can only try to understand themselves; they cannot start from trying to understand God. Here again, he is building on a long tradition of debates about divine inscrutability in Islamic thought, one effect of which has been an acknowledgement of different points of view as equally valid.^58^ He argues that trying to understand the meaning of life and *shari’a*’s role can only be successfully managed if humans start with their own life, their context, and the choices that they can make.^59^ It is in making these choices that humans exert their will, with the full knowledge that they can never have all the resources to judge other human beings in their entirety. That judgment is ultimately the right of Allah. This knowledge builds a deep sense of humility for Ghamidi. It entails not just an active effort to avoid pride^60^ and develop patience^61^ but also to develop a state of being/of the heart (*qalbi kafiyat*) that is traditionally defined as *khushū’*.

*Khushū’* results in complete transformation of the self by tying ones spiritual aspirations with social life such that one becomes “the embodiment of mercy and kind-heartedness” for one’s family, friends, and society at large.^62^ The term holds several meanings but the key connotation of *khushū’* is humility; its etymological root *khasha’a* signifies lowering when applied to looking and speaking.^63^ The relationship of humility with individual piety has received continued attention in Islamic thought and practice to the present day. One reflection of the importance of humility is that even today it forms an important quality a *mufti* (those who can issue a *fatwa* or scholarly ruling and are expected to adjudicate in civil matters) must develop and exhibit in their writings, for instance, by explicitly acknowledging limits to their knowledge.^64^ The expectation is that those who practice it are likely to keep open the door to epistemic humility—to the possibility that their lack of knowledge requires them to consult widely, acknowledge the validity of different positions, and judge others less harshly than they would otherwise.

The deep imprint of humility on a range of Islamic practices including travel in search of knowledge is useful to trace to build what Roxanne Euben has called “countergenealogies” of concepts.^65^ Ghamidi’s use of *khushū’* is not particularly innovative and relies upon a wide circulation of ideas, practices, and terms that mark humility as a key attribute of a pious Muslim. His use is very similar to the use of the term *hayā*’ by the Morocco-based philosopher Abdurrahman Taha to also mean humility and modesty. In his reading of Taha’s ideas, Hallaq suggests that for Taha “humility, modesty, and gratitude are the positive, non-defensive and self-confident modus vivendi of being in the world” that moves humans beyond the “desire to dominate.”^66^ Other modern thinkers such as Syed Qutb have taken humility in pious devotion to Allah to “constitute freedom from enslavement to arbitrary human authority.”^67^ Relying on the wide and deep endorsement of humility in Islamic thought, Ghamidi takes for granted the familiarity of his audience with the notion of *khushū’.*

For Ghamidi, as for Qutb, there is no contradiction between free will and this sense of humility borne through an acceptance of an all-knowing divine. Humans, he argues, have freedom over choosing good or evil. Once a human being decides to act on the right path, Allah helps him. But, he argues, “if the human being decides to suppress his conscience then Allah does not stop him. . . . It is the responsibility of the individual to choose the right path. It is not Allah’s job to keep providing guidance to those who choose not to make any use of it.”^68^ Thus, the ultimate responsibility rests with humans, but this sense of control over one’s life is infused with a deep feeling of humility that, in Ghamidi’s view, discourages the urge to judge others and affords a sense of freedom that is not reliant upon the negation of somebody else’s beliefs and practices.

It is this relationship with the divine that creates social harmony rather than state laws. Ghamidi repeatedly emphasizes that there is a clear difference between the claims that Allah can make on Muslims and the ones that a state—any state, Islamic or not—can make on them. Thus, he formulates a complicated response to a question asked in one of his public appearances.^69^ An audience member asked why Ghamidi, otherwise so moderate and tolerant, supported such barbaric practices as the cutting off of hands as a punishment for theft. Ghamidi responded that the purpose of his own life was to become close to Allah and to bring his understanding of shari’a to a wider audience. It was, he said, evident that Allah has laid down some rules very clearly in the Quran about prayers, *zakat*, and fasting, or more broadly justice, piety, and responsibility toward fellow human beings. These are all matters on which individuals will be judged. While there is a range of options available within shari’a for, say, the crime of theft, such as forgiving the thief or requiring her only to return the goods stolen, the maximum punishments for theft that may seem harsh are nevertheless laid down by Allah. Humans can interpret when to impose the maximum penalties, or *hudud*, in Islamic jurisprudence, but they cannot declare them invalid.^70^

Two critical distinctions he wanted the audience to remember were the following. First, these maximum punishments only apply to Muslims. It is only when Muslims steal and break their pact with Allah that they can be punished in this way. Since non-Muslims did not make this pact with Allah, they are not liable for the maximum punishment.^71^ But the second and more critical issue he highlighted is that the final judgment can only be made by Allah. Reminding his audiences that humans, when in positions to judge others, must bear that thought in mind, Ghamidi implied that while the maximum punishment of cutting off a hand was indeed an option, judges should be humble about their ability to know all the relevant facts and, hence, merciful. He emphasizes the importance of spiritual equality and epistemic humility before Allah where the judge has to remain cognizant of the fact that he too will be judged by a higher authority.

Individual responsibility shot through with humility for him is the scaffolding that ensures sound conduct from oneself in social, political, and economic spheres. He argues that the primary addressee in Islam is the individual.^72^ This notion of the individual is suffused with spirituality and is bound in a close relationship to the divine. Unlike the Islamists, Ghamidi does not argue for political mobilization as a requirement of piety in the modern age.^73^ The expectation of similar pious behavior from others is also minimized. While a morally sound society is, to Ghamidi, a logical consequence if most individuals take their responsibility seriously, any possibility of actively reconstructing society beyond the force of example and some limited *da’wa* (invitation) is, to him, not supported in the Quran. Tolerance here is the consequence of reticence to sit in judgment of others due to a strong sense of one’s own limitations and fallibility, and a shari’a-inspired minimal state.

Critically then Ghamidi’s insistence that he is not a liberal alerts us to differences between his vision and liberal conceptions of tolerance. It is clear that he is not interested in the question of individual freedom. His scheme has the potential for supporting a hegemonic state due to the emphasis he places on obedience to the state and on avoiding conflict.^74^ His underlying assumptions draw sharp distinctions between Muslims and non-Muslims. The Islamization of self and society that Ghamidi envisions is profound because of the emphasis on self-enforcement. With his strict hermeneutic approach, Ghamidi is minimalist regarding political responsibilities but demanding about the minimum criteria of political obedience and individual piety.

## Rethinking Tolerance in Liberalism

Ghamidi’s nonliberal tolerance affords us an opportunity to reconsider the strength of the relationship between liberalism and tolerance. The causal association between individual virtues, social norms, and political institutions for tolerance in liberal thought and practice is not as easy to delineate, as it might seem at first glance. Moreover, the tense relationship between individual autonomy and equality that is a feature of liberal thought, more generally, marks also the liberal conception of tolerance. While some have tried to reconcile the two (important among those are John Rawls and Will Kymlicka), others have moved to claim individual autonomy as the primary focus of liberal tolerance.^75^ Chandran Kukathas, for instance, argues that when liberal thinkers such as Rawls and Kymlicka lose the focus on individual autonomy they become invested in upholding the liberal political order and cannot escape the interest in restructuring minority communities so that they are brought in accord with the majority practice. This, he proposes, “offers insufficient toleration to minority communities. What it evinces, ultimately, is a greater concern with the perpetuation or reproduction of the liberal order, but at the risk of intolerance and moral dogmatism.”^76^ Kukathas proposes a sharp focus only on individual liberty as the remedy to this vacillation between liberty and equality in the proposals by Rawls, Kymlicka, and Iris Marion Young.

While they might disagree with the remedy proposed by Kukathas, other critics have also highlighted problems with the more expansive, and consequently intrusive, vision of liberal tolerance. For instance, state practices geared toward celebrating and/or resolving questions of race, gender, class, and cultural differences inevitably fail to meet the requirements for state neutrality.^77^ For some, the suggestion that a state or a government can be tolerant is nonsense because, “[t]olerance is not only shown exclusively *to* people but also exclusively *by* people.”^78^ Most critically, however, the expansive vision of tolerance raises the question, why tolerance? Why not just rights?^79^

Nevertheless, differently inflected visions of liberalism agree on according importance to individual freedom as a core element of liberal tolerance. The focus on individual autonomy linked to the idea of the freedom of conscience is, of course, deeply embedded in a very specific history of European experience and ideas. Rawls famously understood the Reformation and the emergence of toleration as the foundation upon which modern individual rights have been built. According to this narrative, the religious schism opened by the Reformation led to scepticism and individual questioning.^80^ Once the autonomy of the individual over her own conscience was recognized, the path was paved, so to speak, for the expansion of this principle to other aspects of the individual’s life, including the right to freedom of religious association, right to free speech, and ultimately a general right to freedom or liberty.^81^ Much liberal debate about tolerance is predicated on this, somewhat flawed, reading of history.^82^ Important areas that have received attention from historians but not from influential political theorists include a longer history of toleration as modus vivendi in medieval Europe and not an Enlightenment discovery;^83^ the influence of Islamic ideas and practices of coexistence from 700 years of Muslim rule in Europe;^84^ the immense conflict engendered by the Reformation not just between sects but within them too, as Protestant dogmatism matched alleged Catholic intransigence;^85^ and the role of secular authorities in promoting war and of religious ones in proposing peace, indeed of persecution and toleration, as elements of the same mode of governance.^86^ These questions of historical accuracy have implications for contemporary theorizing that need further attention. As Murphy has argued persuasively regarding the limitations of religiously inspired ideas of freedom of conscience in addressing contemporary identity politics, “we should not ask our concepts to perform impossible tasks.” When theorists such as Rawls rely on an inaccurate historical account of the emergence of liberal tolerance they run the danger of misrepresenting present day concerns as well as historical figures central to the tradition and, most importantly, misunderstanding the nature of both problems and possible solutions.^87^

Notwithstanding such important questions and the historical scholarship that destabilizes the established narrative about the emergence of tolerance in the liberal conception, prominent liberal theorists continue to rely on this reified history to conceive of tolerance through its relationship with individual liberty. Indeed, liberalism conceptualized as a unified tradition of thought is itself a recent and still slippery being. It was only in the twentieth century that the newly emerging academic discipline of political theory, “as the theory of the state and the history of political ideas . . . perhaps best seen as an American product” facilitated the emergence of liberalism as singular tradition of thought with a thematic interest in pluralism.^88^ John Locke, commonly regarded today as a foundational thinker in the liberal tradition, particularly with regard to the question of tolerance, was for several centuries ignored in British political philosophy; the reasons and timing of his emergence as such a figure in contemporary liberalism varied between Britain and America.^89^

Locke’s ideas then bore a heavy burden, moving much beyond his original propositions. Locke proposed a deeply, religiously inspired version of toleration when he argued for limits to the magistrate’s powers in regulating individual conscience. Rather than arguing against the general use of force by the state to impose a set of beliefs, which is how Locke’s argument is often read today, he was concerned primarily with clarifying the reach of royal authority regarding a narrow set of Christian beliefs and was elsewhere prejudiced along racial and religious lines. He is unsympathetic, indeed hostile, toward wide-ranging freedom of thought and expression^90^ and was not making a positive case for diversity and difference; there is no real concern with whether “there is anything *morally* wrong with intolerance, or a sense of any deep concern for the *victims* of persecution. . . . ”^91^

Similarly, while J. S. Mill was an important influence in British liberal thinking, his ideas on the question of minorities were not widely taken up; if anything, he was swimming against the mainstream of European thought on the question of race and biological determinism.^92^ Nevertheless, in contrast to Locke, Mill’s ideas are structured much more robustly around a concern for the minority individual or group. For Mill, diversity is important not as “an aesthetic preference”^93^ but because it is intrinsic to human life, a necessary precondition of human flourishing and political progress.^94^ Mill certainly provides greater resources for contemporary liberal thinkers. There remain, however, important concerns with regard to the reliance upon a specific construction of rationality as an exclusionary mechanism within Mill’s conception of freedom^95^ and of harm as an ambiguous notion that makes neutrality impossible.^96^

More importantly, for our purposes here it is useful to note that although a concern for minority individuals and groups has been a feature of *liberal thinking*, it was not a central or even a prominent feature of *liberal polities* until the end of the twentieth century. Significantly, while the thrust toward homogeneity within European and American intensified with the increasing dominance of nationalism and racism through the nineteenth and early twentieth centuries, the question of minority rights became an important tool in extending colonial control overseas.^97^ It took the rise of communism and Nazism to solidify the postwar association of tolerance with liberalism, as it was tied to the attempt at defining liberalism in opposition to monolithic impulses in communism and totalitarianism. The postcolonial theorist David Scott reminds us that,

prior to World War II, the liberal democratic claim on behalf of cultural diversity was a comparatively weak one. In the climate of aggressive assimilationism that followed World War I, . . . within the United States there was little patience for cultural diversity or minorities. However, the concerted battle against the Nazi state in Germany and the Communist one in the Soviet Union gave rise to a new preoccupation with liberal democracy as precisely the embodiment of pluralism and cultural tolerance.^98^

At the very least, and contrary to the now dominant political discourse, a linear trajectory of ever-increasing tolerance in Europe since the Reformation and in conjunction with liberal ideas is historically suspect. Liberal ideas and actual liberal practice from the seventeenth to the twentieth century have not knitted together as neatly as important liberal theorists such as Rawls seem to have assumed. It is only toward the end of the twentieth century that tolerance was theorized as a positive recognition of difference.^99^ This more expansive definition of tolerance is linked to the question of multiculturalism at a time when it became “the central problematic of liberal democratic citizenship: as Third World immigration threatened ethnicized identities of Europe, North America and Australia; as indigenous peoples pursued claims of reparation, belonging and entitlement; as ethnically coded civil conflict became a critical site of international disorder; and as Islamic religious identity intensified and expanded into a transnational political force.”^100^

Ghamidi highlights a different vision of tolerance that is not concerned directly with the question of individual freedom. He proposes strong allegiance to the state at the same time as dramatically curtailing the state’s powers in shaping individual and communal life. By drawing upon the popularity of the notion of *khushū’* as well as a shari’a-inspired state minimalism, Ghamidi articulates a mode of peaceful coexistence that moves away from debates about legal rights, individual freedom, and the state to privilege individual humility and duty.

Tolerance is entirely and uniquely liberal if by it we mean an overriding concern for individual freedom preserved in a legal framework. Enshrined in international legal regimes and promoted by powerful political actors today, such a vision of tolerance is very much the product of a specific social history and particular institutional arrangements that developed in Europe and led to liberalism. Ongoing debates demonstrate that operationalizing the ideal is a challenge in real life, particularly in contexts where historical experiences differ from Europe. For instance, Jeff Spinner-Halev has perceptively noted the limitations a liberal vision of religious freedom places in contexts where religion is not conceived of as interiorized belief but ritualized social interaction and practice.^101^ He notes the difficulty that Indian courts, within a broadly liberal regime, face in adjudicating in cases where the liberal ideal of equality for Dalits, placed at the lowest rung in the Hindu caste system, clashes with claims of the upper caste Hindus that rituals of purity and separation form an integral part of their religion. Upper caste Hindus’ freedom of individual conscience and protection from interference in their religion by the state clashes directly with the Dalit right to equality.

We then have to be alert to the possibility that the focus on legal rights and individual freedom of conscience might have the potential for creating new and sometimes very intransigent divisions. The centrality of individual freedom of conscience in debates about minorities belies the reliance upon European experiences that may not travel well everywhere nor might they provide adequate foundations for building robust polities. In a perceptive analysis of the ways in which Locke’s ideas are (mis)read by contemporary theorists, Timothy Stanton proposes that at the heart of liberal conceptions of freedom and authority lies an unresolved dilemma, “how a determinate pattern of legitimately authoritative political institutions and offices can be derived from the autonomous will and agreement of individuals—and nothing else—without succumbing to the centrifugal force of philosophical anarchism.”^102^ Within the Islamic tradition this dilemma is addressed through a different set of assumptions about the centrality of duty in governance and citizenship, the value of humility for the individual expressing itself in a reticence to judge, and the implications of these in allowing peaceful political engagement as well as coexistence with the “other.”

Rethinking the relationship between tolerance and liberalism also raises an important question about the role of secularism in contemporary politics. Secularism has normative value in liberal theory only insofar as it is seen as the means to promote tolerance. Ghamidi, a particularly lucid commentator, articulates just one of many nonliberal conceptions of tolerance that bypasses the state management of religion that secularism entails.^103^ Anthropologists and historians of Ghamidi’s native South Asia have noted the depth and longevity of nonliberal tolerance in the region. In particular, the cross-religious symbolism of Sufi shrines and the many everyday practices of peaceful coexistence in the highly diverse multireligious, multiethnic, and multilingual megacities of South Asia have received much attention.^104^ The liberal colonization of tolerance needs questioning precisely because other, potentially valuable forms of tolerance may be eroded by a legalistic discourse of tolerance resting on individual freedom of conscience. At the very least, Ghamidi’s nonliberal tolerance opens up many questions for consideration about the relationship between liberalism and tolerance, a relationship much more recent and unstable than often assumed. Sustained, critical, and detailed engagement with nonliberal tolerance is an urgent political and intellectual project.

## Notes

1 Stephen Kautz, “Liberalism and the Idea of Toleration,” *American Journal of Political Science* 37/2 (May 1993): 610–32, 610.

2 Chandran Kukathas, *The Liberal Archipelago: A Theory of Diversity and Freedom* (Oxford: Oxford University Press, 2003), 24.

3 As with many popular ideas tolerance remains a “philosophically elusive concept” (David Heyd, *Toleration: An Elusive Virtue* [Princeton: Princeton University Press, 1996], 2). To start with, there is confusion about the difference between tolerance and toleration. Often the terms are used interchangeably. Nevertheless, there have been attempts at precision (Michael Walzer, *On Toleration* [New Haven: Yale University Press, 1997] xi, 10–11); see also Andrew Murphy, “Tolerance, Toleration, and the Liberal Tradition,” *Polity* 29, no. 4 (Summer, 1997): 595. Others see toleration as a virtue, albeit one that is paradoxical (Susan Mendus, *Toleration and the Limits of Liberalism* [London: Macmillan, 1989], 20). There is further debate about whether it is a moral attitude, a political value, or an institutional arrangement. (Susan Mendus and John Horton, eds., *Aspects of Toleration* [London: Methuen, 1985]; Peter Jones, “Making Sense of Political Toleration,” *British Journal of Political Science* 37, no. 3 [July 2007]: 383–402).

4 John Rawls, “Justice as Fairness: Political not Metaphysical,” *Philosophy and Public Affairs* 14 (1985): 223–51; Joseph Raz, “Liberalism, Autonomy and the Politics of Neutral Concern,” *Mid-West Studies in Philosophy* 7 (1982): 89–120, and also, “Autonomy, Toleration and the Harm Principle,” in *Justifying Toleration: Conceptual and Historical Perspectives*, ed. Susan Mendus (Cambridge: Cambridge University Press, 1988); Jeremy Waldron, “Locke: Toleration and the Rationality of Persecution,” in *Justifying Toleration: Conceptual and Historical Perspectives*, ed. Susan Mendus (Cambridge: Cambridge University Press, 1987): 61–86; Will Kymlicka, *Multicultural Citizenship: A Liberal Theory of Minority Rights* (Oxford: Oxford University Press, 1996); Mendus, *Justifying Toleration*; Kautz, “Liberalism”; Murphy, “Tolerance, Toleration”; Kukathas, *Liberal Archipelago*.

5 Mendus, *Toleration and the Limits*, 3.

6 Delawar Hussain, “South Asia’s Welcoming Sufi Shrines,” *The Guardian*, October 31, 2010, https://www.theguardian.com/commentisfree/2010/oct/31/sufi-shrines-south-asia.

7 “Attitudes to Same-Sex Relationships Around the World: Sub-Saharan Africa is more Liberal than Asia,” *The Economist*, November 2, 2017, https://www.economist.com/graphic-detail/2017/11/02/attitudes-to-same-sex-relationships-around-the-world.

8 Shahan Mufti, “The Fundamentalist Moderate,” *Boston Globe*, July 22, 2007, http://www.boston.com/news/globe/ideas/articles/2007/07/22/the_fundamentalist_moderate/?page=full.

9 Mohammed Khalid Masud, “Rethinking Sharia: Javed Ahmad Ghamidi on Hudud,” *Die Welt des Islams* 47, no. 3-4 (2007): 356–375, 356.

10 Sadaf Aziz, “Making a Sovereign State: Javed Ghamidi and ‘Enlightened Moderation,’” *Modern Asian Studies* 45, no. 3 (2011): 598–99.

11 See, for instance, “What is Liberal Islam?” in *Friday Times*, a Pakistani newspaper, http://www.thefridaytimes.com/beta2/tft/blog.php?blogstory=4), and “Islamic Scholar Attacks Pakistan’s Blasphemy Laws,” in the UK-based *The Guardian*, http://www.theguardian.com/world/2011/jan/20/islam-ghamidi-pakistan-blasphemy-laws, both accessed December 5, 2019.

12 Javed Ahmed Ghamidi, “*Kia Islam Liberal Hai?”* (Is Islam Liberal?), *Dunya Television*, March 4, 2018, https://www.youtube.com/watch?v=N7U0oHCRNOU.

13 Fazlur Rahman, quoted in Charles Kurzman, *Liberal Islam: A Sourcebook* (Oxford: Oxford University Press, 1998), 11.

14 Ole Peter Grell and Bob Scribner, eds., *Tolerance and Intolerance in the European Reformation* (Cambridge: Cambridge University Press, 1996), 5–7. John Dunn argues that, for Locke, toleration is not to be extended at the expense of sovereignty (“The Claim to Freedom of Conscience: Freedom of Speech, Freedom of Thought, Freedom of Worship?” in *From Persecution to Toleration: The Glorious Revolution and Religion in England*, eds. Ole Peter Grell, Jonathan I. Israel, and Nicholas Tyacke [London: Oxford University Press, 1991], 185–186).

15 Andrew March, *Islam and Liberal Citizenship: The Search for an Overlapping Consensus* (Oxford: Oxford University Press. 2009), 19.

16 I am grateful to a reviewer for raising this issue. For critiques of studies privileging compatibility between liberalism and Islam, see Saba Mahmood, *Religious Difference in A Secular Age: A Minority Report* (Princeton: Princeton University Press, 2015); Roxanne Euben, “Making the World Safe for Compatibility,” *Political Theory* 38, no. 3 (2010): 424–41; Humeira Iqtidar, “Searching for ‘Tolerance’ in Islamic Thought,” in *Handbook of Comparative Political Theory*, eds. Leigh Jenco, Megan Thomas, and Murad Idris (Oxford: Oxford University Press, 2019), 525–45.

17 David Scott, “The Aftermaths of Sovereignty: Postcolonial Criticism and the Claims of Political Modernity,” *Social Text* 48 (1996): 5, 21.

18 Mufti Faisal Khurshid Japanwala, *Ghamidi Sahib Ulema Ki Nazar Men* (Karachi: Time Club, 2008).

19 These are detailed in Ghamidi, *Mizan* (Lahore: Al-Mawrid Institute for Education and Research, 2018 [1990]), 15–66. For a concise overview, Asif Iftikhar, “Jihad and the Establishment of Islamic Global Order: A Comparative Study of the Worldviews and Interpretative Approaches of Abü al-A’la Mawdüdi and Javed Ahmad Ghamidi” (MA thesis, Institute of Islamic Studies, McGill University, 2004). Ghamidi’s methodological systematization is not without implications about the vibrancy of debate within Islamic thought (Humeira Iqtidar, “Redefining Tradition in Political Thought,” *European Journal of Political Theory* 15, no. 4 [2016], 424–44).

20 Javed Ghamidi, *Islam: A Comprehensive Introduction* (Lahore: Al-Mawrid Institute for Education and Research 2010), 452.

21 Ibid., 454–55.

22 Javed Ghamidi, *Muqamat* (Lahore: Al-Mawrid Institute for Education and Research, 2017 [2008]), 147–68.

23 For the centrality of justice in an ideal Islamic polity see Majid Khaduri, *The Islamic Conception of Justice* (Baltimore: Johns Hopkins University press, 1984); Linda Darling, *A History of Social Justice and Political Power in the Middle East: The Circle of Justice from Mesopotamia to Globalization* (London: Routledge, 2013); Lawrence Rosen, *The Justice of Islam: Comparative Perspectives on Islamic Law and Society* (New York: Oxford University Press, 2009).

24 Ghamidi, *Islam*, 456.

25 Wael Hallaq, *The Impossible State: Islam, Politics and Modernity’s Moral Predicament* (New York: Columbia University Press, 2014), 48–60; Albert Hourani, *Arabic Thought in the Liberal Age* (Cambridge: Cambridge University Press, 1983[1962]), 161–93; Muzaffar Alam, *The Languages of Political Islam: India 1200-1800* (Delhi: Permanent Black, 2004), 46–76.

26 Ghamidi, *Islam*, 462.

27 In the broad thrust of his arguments Ghamidi refutes Maududi’s support for state-imposed Islamic piety. However, Ghamidi never directly opposes Maududi by name and always mentions him with deep respect. The intellectual debt that Ghamidi most frequently acknowledges is to Maulana Islahi, who was also a founding member of the JI but left the party after a decade.

28 Ghamidi, *Islam*, 464, see also *Mizan*, 495–98.

29 Ghamidi, *Islam*, 460.

30 Founded in late nineteenth-century colonial India, the Ahmadiyya movement is controversial because of doctrinal differences with Sunni beliefs regarding the finality of prophethood.

31 “Pakistan Islami riyasat nahin hai?” Youtube video, https://www.youtube.com/watchv=eZ4v1vu8Aj4, accessed September 01, 2020.

32 Also Ghamidi, *Muqamat*, 191–95.

33 Ibid., 235–38.

34 There is debate about the status of three Jewish tribes after the initial peace agreement with them. Two tribes were expelled and male members of the third were executed, all on suspicion of colluding with external enemies. See Anver Emon, *Religious Pluralism and Islamic Law: Dhimmis and Others in the Empire of Law* (Oxford: Oxford University Press, 2012, 46–54) for details. Ghamidi’s interpretation implies that the key principle was equality in return for loyalty to the state of Medina. The tribes did not lose their status just because they were a different religion but because they reneged on their treaty promise.

35 Ghamidi, *Islam*, 461.

36 Arthur Stanley Tritton, *The Caliphs and their Non-Muslim Subjects* (London, Oxford University Press, 1930), 216–26.

37 See Emon, *Religious Pluralism*, 100–2, for a discussion about variation in rates and possible uses of *jizya* in financing the early Islamic imperial expansion.

38 Ghamidi, *Islam*, 460.

39 Milka Levy-Rubin, *Non-Muslims in the Early Islamic Empire: From Surrender to Coexistence* (Cambridge: Cambridge University Press, 2011).

40 For instance, in terms of economic participation, comparing the Islamic world with Europe, Mark Cohen, “The ‘Golden Age’ of Jewish Muslim Relations: Myth and Reality,” in *A History of Jewish-Muslim Relations: From the Origins to the Present Day*, eds. Abdelwahab Meddeb, Benjamin Stora (Princeton: Princeton University Press 2013), 32–33, suggests that Jews and Christians in the Islamic world were not ghettoized into specific economic professions as Jews had been in Europe during this period.

41 Emon, *Religious Pluralism*, 142.

42 Ghamidi, however, refutes these associations with secularism, stating “I am neither an Islamist, nor a secular.” in “Islamic scholar” *The Guardian*.

43 This contemporary discourse of tolerance is, of course, in some contrast to toleration, which comes more clearly embedded in a complex of power imbalance. Toleration has very specific religious origins in the “concession of liberty to those who dissent in religion” in the European context (Henry Kamen, *The Rise of Toleration* [London: Weidenfeld and Nicholson, 1967], 7). Its expansion to include freedom of thought, speech, and expression, as well as equality of citizenship rights, forced an elasticity upon the original formulation that may not be philosophically sustainable within the framework of its original authors such as Locke (Dunn, “Claim to Freedom”) or Erasmus (Istvan Bejczy, “*Tolerantia*: A Medieval Concept,” *Journal of the History of Ideas* 58 [1997]: 365–84), or its founding context (Jones, “Making Sense,” 384–86).

44 For example, *Muqamat*, 187–88, 198.

45 Lloyd Ridgeon, ed. *Islam and Religious Diversity* (Abingdon: Routledge 2012); Shahab Ahmed, *What is Islam?* (Princeton, Princeton University Press, 2016), chapter 1; Emon, *Religious Pluralism*, 141–43.

46 Wael Hallaq, *Sharī’a: Theory, Practice, Transformations* (Cambridge: Cambridge University Press, 2009), and *Authority, Continuity and Change in Islamic Law* (Cambridge: Cambridge University Press, 2001).

47 Ridgeon, *Religious Diversity*; David Gilmartin and Bruce Lawrence, eds. *Beyond Turk and Hindu: Rethinking Religious Identities in Islamicate South Asia* (Gainseville: University Press of Florida, 2003); Cohen, *Golden Age*.

48 Talal Asad, *Formations of the Secular: Christianity, Islam, Modernity* (Stanford: Stanford University Press, 2003).

49 Ovarmir Anjum, “Dhimmi Citizens: Non-Muslims in the New Islamist Discourse,” *ReOrient* 2, no. 1 (2016), 33.

50 See Hallaq, *Impossible State*, 3–18, 156–62; Rosen, *Justice of Islam*, 20–23.

51 It is worth noting that many Protestant reformers were as keen on taking over the state as the Catholic Church had been, turning the Reformation into a very long and bloody process (Kamen, *Rise of Toleration*, 22–55, 71–73, 103–6; Grell and Scribner, *Tolerance and Intolerance*).

52 Mohammed Qasim Zaman, *The Ulama in Contemporary Islam: Custodians of Change* (Princeton: Princeton University Press, 2007).

53 Khaled Abou El Fadl, *The Place of Tolerance in Islam* (Boston: Beacon Press, 2002).

54 Fazlur Rahman, *Revival and Reform in Islam: A Study of Islamic Fundamentalism* (Oxford: One World, 1999); March, *Overlapping Consensus*, 207–58.

55 Paul Powers, *Intent in Islamic Law: Motive and Meaning in Medieval Sunni Fiqh* (Leiden: Brill, 2006), 9–23.

56 Interestingly, while agreeing with the dominant view about the emergence of humanism during the Renaissance as a crucial step in the process of individual liberation, Larry Siedentop (*Inventing the Individual: The Origins of Western Liberalism* [London: Penguin, 2014], 337–43) subtly transforms the narrative about humanism. He contends that the Italian humanists went back to ancient Greece using it as a “kind of quarry” for ideas and practices, and though they were explicitly hostile to the church as an institution, they ended up combining Greek ideas with Christian ideas that the church had naturalized over the previous centuries to support the rise of the “fundamental feature of modernity . . . an individuated model of society” (336). Humanism for Siedentop is profoundly infused with Christianity.

57 Mark Button, “‘A Monkish Kind of Virtue’? For and Against Humility.” *Political Theory* 33, no. 6 (Dec. 2005), 842.

58 Hallaq, *shari’a*, 65–78; John Walbridge, “The Islamic Art of Asking Questions: *‘Ilm al-Ikhtilāf* and the Institutionalization of Disagreement,” *Islamic Studies* 41, no. 1 (Spring, 2002), 69–86.

59 He provided a particularly cogent explanation at a public event organised by Al-Mawrid Australia on October 11, 2015 (accessed December 5, 2019), “Allah’s knowledge and Freedom of Choice”, YouTube video, https://www.youtube.com/watch?v=qj-Ow3ZR-9M.

60 Ghamidi, *Mizan*, 95–96, 238–41.

61 Ibid., 250–54.

62 Ibid., 255.

63 ‘Amir Hayder et al., eds. *Lisan al-’Arab*, vol. 8 (Beirut: Dal Kutub al-illmiyya, 2009), 84–85.

64 Hussain Agrama, “Ethics, tradition, authority: Toward an anthropology of the fatwa,” *American Ethnologist* 37, no. 1 (2010), 12.

65 Roxanne Euben, *Journeys to the Other Shore: Muslim and Western Travelers in Search of Knowledge* (Princeton: Princeton University Press, 2006), 178–79.

66 Wael Hallaq, *Reforming Modernity: Ethics and the New Human in the Philosophy of Abdurrahman Taha* (New York: Columbia University Press, 2019), 268–70.

67 Roxanne Euben, “Humiliation and the Political Mobilization of Masculinity,” *Political Theory* 43, no. 4 (2015), 512.

68 “Top ten questions and answers 2016 with Javed Ghamidi” Youtube video, accessed February 6, 2019, https://www.youtube.com/watch?v=umeRs1Xpsn0.

69 “How can a rational person support sharia when it is so barbaric?” Youtube video, accessed February 6, 2019, https://www.youtube.com/watch?v=Axsj3eEUBd0.

70 Maximum penalties for certain crimes are called *hudud* in Islamic jurisprudence. General Zia attempted to include them into the country’s penal code and faced resistance from human rights activists and from some Islamic scholars, including Islahi.

71 “How can a rational person support sharia” Youtube video. See also Ghamidi, *Muqamat*, 238–39.

72 Ghamidi, *Muqamat*, 193.

73 cf. Syed Abul A’ala Maududi, *Al-Jihad fi sabil Allah* (Lahore: Idara M’arafat-e-Islami, 2007 [1930]).

74 Sadaf Aziz, “Making a Sovereign State,” 623–27.

75 Raz, “Autonomy, Toleration”; Kukathas, *Liberal Archipelago*.

76 Kukathas, *Liberal Archipelago*, 125–26.

77 Mendus, *Toleration and the Limits*, 135–45.

78 Heyd, *Toleration*, 15.

79 Wendy Brown, *Regulating Aversion: Tolerance in the Age of Identity and Empire* (Princeton: Princeton University Press), 2006.

80 cf. Richard Tuck, “Scepticism and Toleration in the Seventeenth Century,” in *Justifying Toleration: Conceptual and Historical Perspectives*, ed. Susan Mendus (Cambridge: Cambridge University Press, 1988), 21–36.

81 cf. Waldron, *Toleration and Rationality*; Dunn, “Claim to Freedom”; Timothy Stanton, “Authority and Freedom in the Interpretation of Locke’s Political Theory,” *Political Theory* 39, no. 1 (2011): 6–30.

82 Jeffrey Collins, “Redeeming the Enlightenment: New Histories of Religious Toleration,” *Journal of Modern History* 81, no. 3 (September 2009), 632–35; Andrew Murphy, *Conscience and Community: Revisiting Toleration and Religious Dissent in Early Modern England and America* (Philadelphia: Pennsylvania State University Press, 2001), 11–24; Teresa Bejan, “Evangelical Toleration,” *The Journal of Politics* 77, no. 4 (2015), 1103–14.

83 Bejczy, “*Tolerentia*.”

84 Salvatore Armando, “The Euro-Islamic Roots of Secularity: A Difficult Equation,” *Asian Journal of Social Science* 33, no. 3 (2005), 412–37.

85 Kamen, *Rise of Toleration*; Grell and Scribner, *Tolerance and Intolerance*.

86 Collins, *New Histories*, 614–15.

87 Murphy, *Conscience and Community*, 292.

88 John Gunnell, *The Descent of Political Theory: Genealogy of an American Vocation* (Chicago: University of Chicago Press, 1993), 56, 105–15; see also, Duncan Bell, “What is Liberalism?” *Political Theory* 42, no. 6 (2014), 682–715.

89 Bell, “Liberalism,” 693–704.

90 Stanton, “Authority and Freedom”; Dunn, “Claims to Freedom.”

91 Waldron, *Toleration and Rationality*, 85 (italics in original).

92 Georgios Varouxakis, *Mill on Nationality* (London: Routledge, 2002), 17–29, 39–50.

93 Mendus, *Toleration and Limits*, 51.

94 J. S. Mill, *On Liberty* (Kitchner: Batoche Books, 2001[1859]), 61–68.

95 Uday Mehta, *Liberalism and Empire: A study in Nineteenth Century British Liberal Thought* (Chicago: University of Chicago Press, 1999), 31–37, 97–106.

96 Mendus, *Toleration and Limits*, 120–42.

97 Saba Mahmood, *Religious Difference*; Mark Mazower, *No Enchanted Palace: The End of Empire and the Ideological Origins of the United Nations* (Princeton: Princeton University Press, 2009).

98 David Scott, “Culture in Political Theory” *Political Theory* 31, no. 1 (2003), 109.

99 Kymlicka, *Multicultural citizenship*, 152–68; Kautz, *Liberalism and the Idea*; Karl-Otto Apel, “Plurality of the Good? The Problem of Affirmative Tolerance in a Multicultural Society from an Ethical Point of View,” *Ratio Juris* 10 (1997), 199–212.

100 Brown, *Regulating Aversion*, 2.

101 Jeff Spinner-Halev, “Hinduism, Christianity and Liberal Religious Tolerance,” *Political Theory* 33, no. 1 (2005), 28–57.

102 Stanton, “Authority and Freedom,” 11.

103 See, in particular, his essay, “*Islam aur riyasat: aik jawabī biyan*,” in *Muqamat*, 196–209.

104 An exemplary study of tolerance practiced by the multireligious citizens of Malerkotla due to their association with a local shrine is by Anna Bigelow, *Sharing the Sacred: Practicing Pluralism in Muslim North India* (Oxford: Oxford University Press, 2010).

